# Motivations, Barriers, and Behaviors Related to Obtaining and Discussing Family Health History: A Sex-Based Comparison Among Young Adults

**DOI:** 10.3389/fpubh.2015.00249

**Published:** 2015-11-20

**Authors:** Matthew Lee Smith, Christopher E. Beaudoin, Erica T. Sosa, Jairus C. Pulczinski, Marcia G. Ory, E. Lisako J. McKyer

**Affiliations:** ^1^Department of Health Promotion and Behavior, College of Public Health, The University of Georgia, Athens, GA, USA; ^2^Department of Health Promotion and Community Health Sciences, Texas A&M Health Science Center, School of Public Health, College Station, TX, USA; ^3^Department of Communication, Texas A&M University, College Station, TX, USA; ^4^Department of Kinesiology, Health and Nutrition, University of Texas – San Antonio, San Antonio, TX, USA

**Keywords:** family history, health communication, family interaction, sex-based differences

## Abstract

**Background:**

Genetic predisposition is a risk factor for many chronic diseases, yet little is known about the frequency in which college students seek out their family health history or with whom they communicate relevant information.

**Purpose:**

This study examines motivations and barriers associated with obtaining one’s family health history and discussing it with others.

**Methods:**

Data were analyzed from 625 college students using an internet-delivered questionnaire, which comprised of questions about intentions and motivations to obtain and share family health history as well as barriers encountered when obtaining family health history. Responses were bifurcated by participants’ sex. Chi-squared and *t* statistics were used to identify response differences by sex.

**Results:**

Females were significantly more likely than males to be motivated to obtain their family health history, and more likely to have shared their family health history with others; state that they would share their family health history with others; and express a preference for sharing their family health history with a wider range of people.

**Discussion:**

Educational interventions and improved student health services could be effective mechanisms to increase college students’ knowledge, awareness, and perceived importance of obtaining their family health history.

## Introduction

Family health history is a risk factor for two of the leading causes of death, heart disease (1) and cancer (2). The importance of family health history has been identified as a critical element in helping to identify patients’ risk of developing disease (3). Yet, the general population reports infrequent behaviors associated with obtaining their family health history, which is especially true among young adults (4). This is particularly troublesome considering that adolescence is a time when life-long health behaviors are established (5). Thus, undesirable health behaviors coupled with an unknown family history may increase the risk for chronic disease and delay medical visits and screenings for early disease detection, which can exacerbate negative health consequences later in life.

Previous studies examining risk perceptions of sex-based disease among college students have focused more on the influence of family history on disease development, and less on the factors associated with obtaining family health history (6). More broadly, research has taken different sides on the subject of whether sex differences matter for communication and information-seeking processes (7, 8), with recent research highlighting sex as a strong predictor of online health information seeking (9, 10). Research also has revealed an ill-founded optimistic bias among individuals with a family history of disease (11), which translates into a false sense of security and beliefs that “it won’t happen to me.” Although, when young adults perceive themselves to be susceptible to disease, the sex-based differences in these perceptions are underestimated and do not represent the actual epidemiological prevalence rates of disease (12). For example, young women continue to fear cancer more than heart disease across various points of the life course (12–14), despite heart disease being the leading cause of death among Americans of both sexes.

What has been missing in the literature is an examination that goes beyond the perceived importance of family health history. There is a lack of knowledge about the types of people college students discuss – and are willing to discuss – their family health history with, what would motivate them to do so, and what barriers and benefits may exist when attempting to acquire such information. Although it is important for college students to discuss family health history with physicians, questions remain about whether they would share such information with physicians, and what circumstances would promote family health history discussions. And, even less is known about how these behaviors may differ by sex, despite such information being useful to tailor health prevention campaigns for sex-specific diseases such as breast cancer.

Research in this area can not only help fill these practical gaps in the literature but can also assist to build upon previous theoretical research investigating what types of people use health information and why they do so. Such theoretical goals have a basis in previous literature about health information seeking (15, 16), the Health Belief Model (HBM) (17), and uses and gratifications (18). Literature in these separate streams overlap when it comes to postulating that health behaviors, including seeking out and using health information, are a function of a person’s motivation, beliefs, and personal characteristics (15, 19). In this light, the current study considers people’s interpersonal discussion about their family health history, their motivation and perceived benefits and barriers for having such discussion, and how these family health-related interactions may vary between men and women.

With this general theoretical framework, the purposes of this descriptive study were to: (1) describe college students’ family health discussions in terms of prior and future discussions and preferences for particular interactions; (2) identify the factors related to family health history discussions, with special attention to sex-based differences; and (3) examine the perceived benefits of and barriers to obtaining one’s family health history.

## Materials and Methods

### Participants and Procedures

The *Finding Roots: Exploring Your Family History* study (12) investigated college students’ knowledge, perceptions, and behaviors pertaining to their family health history and chronic diseases (i.e., cancer, heart disease, diabetes, and obesity/overweight). Data were collected from 703 college students over the age of 18 years using internet-based data collection methods. Participants were recruited from one Texas University in the United States. Various recruitment methods were used in this study, which included campus-wide postings of recruitment flyers and open-access computers, and e-mail messages to academic advisors. The survey was available for an 8-week period. Because of the diverse strategies to recruit participants from an array of channels, the exact response rate could not be determined (12, 13, 20, 21). Participation in this study was voluntary, and participants could withdraw from the study at any time. No identifying information was collected, thus confidentiality was maintained. All components of the study were approved by the Texas A&M University Institutional Review Board.

### Instrument

Participants were surveyed using an internet-based instrument, which consisted of 60 items and included Likert-type scales, checklists, and close-ended response formats. Instrument items were created by the study investigators and were derived using focus group methodology. Detailed information about the *Finding Roots: Exploring Your Family History* study can be found elsewhere (12, 13, 20, 21). Participants took approximately 15 min to complete the questionnaire.

### Measures

#### People with Whom to Discuss Family Health History

Participants were asked to identify with whom they *have discussed* their family health history, with whom they *would discuss* their family health history, and with whom they *prefer to discuss* their family health history. For each aspect of family health history interpersonal discussion (i.e., have, would, prefer), the participants were asked to select “all that apply” from a list of 10 types of individuals (i.e., parents, siblings, extended family, friends/peers, physician/healthcare provider, teacher/educator, religious figure, stranger, other, no one). For all three aspects, responses were individually compared by sex for each type of individual and the total number of responses endorsed by the participant within that particular aspect.

#### Factors Influencing Family Health History Discussions

Participants were asked to identify factors that would influence them to discuss their family health history, their perceived benefits of obtaining their family health history, and their perceived barriers to obtaining their family health history. The items on the factors that influence interpersonal discussion have a general basis in research on uses and gratifications (18) and Comprehensive Model of Information Seeking (CMIS) (15, 19), whereas the items on perceived benefits and barriers have a general basis in literature on HBM (17) and CMIS (15, 16). For each factor (i.e., have discussed family health history because, would discuss family health history because, would be motivated to obtain family health history because, perceived benefits of obtaining family health history, barriers obtaining family health history in the next 3 days), the participants were asked to select “all that apply” from a list of response choices (see Table 3). A 3-day time parameter was assigned to this set of items to measure participants’ perceived ability to obtain such information in a timely manner. For example, if an individual was to experience symptoms consistent with a chronic condition and thus schedule a screening appointment with a physician, what is the reality that the individual could actually obtain the desired information quickly? This measures not only the participants’ ability to get the information if needed but also indicates the proximity of health information-related channels and the degree to which each item was a barrier.

#### Personal Characteristics

To describe the demographic and health characteristics of college student participants, study variables included age; race/ethnicity; whether or not the participant had health insurance; whether or not the participant had a regular physician; number of times the participant was asked by a physician’s office to update family history records; who participants’ perceived was responsible for initiating family health history discussions in healthcare settings (i.e., self, physician, or equal responsibility between the two); number of family members diagnosed with a chronic condition (i.e., parent, grandparent, older/younger sibling, aunt/uncle); and number of non-family members diagnosed with a chronic condition (i.e., friends, friends’ parents, classmates, co-workers, other). Furthermore, sex was used as the primary demographic for testing differences in the aforementioned two sets of variables: people with whom to discuss family health history and factors influencing family health history discussions. Using sex in this manner is underpinned by previous research that has signified the presence of sex differences in health communication and information-seeking processes (8–10).

### Data Analysis

All statistical analyses for this descriptive study were performed using SPSS (version 20). Of the 703 college students who completed the survey with instrument, 78 participants self-reported being diagnosed with one of the health conditions examined in this study (e.g., heart disease, diabetes, cancer, or being overweight/obese) and were removed from analyses to limit bias. The resulting analytic sample consisted of 625 participants. Frequencies were generated for all participants and individually based on sex. Tests of association between participants’ sex were based on Pearson’s chi-squared statistics for categorical response choices. Mean differences between sexes were based on *t*-test statistics for continuous response choices (i.e., the summed number of response choices endorsed for each aspect and factor independently).

## Results

### Sample Characteristics

Sample characteristics of study participants are presented in Table 1. Approximately 62.6% (*n* = 391) of respondents were females. The majority of participants reported being between the ages of 20 and 24 years (66.08%), being non-Hispanic white (73.1%), having health insurance (89.92%), and having a regular physician (67.4%). Most participants (93.8%) reported being asked by their physician’s office to update their family health history one or more times. Approximately 42% of participants perceived that it was their responsibility to initiate family health history discussions in a healthcare setting compared to 21.4% who perceived the responsibility was that of the physician and 30.6% who perceived the responsibility was equal between them and the physician.

**Table 1:** **Sample characteristics by sex (*n* = 625)**.

	Male (*n* = 234)	Female (*n* = 391)	Total (*n* = 625)	X^2^/t	*p*
**Age**					
18 years old	23 (9.8%)	37 (9.5 %)	60 (9.6 %)	5.252	0.629
19 years old	56 (23.9%)	72 (18.4 %)	128 (20.48 %)	
20 years old	62 (26.5%)	127 (32.5 %)	189 (30.24 %)	
21–24 years old	86 (36.8%)	138 (35.3 %)	224 (35.84 %)	
25+ years old	7 (3.0%)	17 (4.3 %)	24 (3.84 %)	
**Race/Ethnicity**					
Non-Hispanic White	157 (67.09%)	300 (76.73 %)	457 (73.1 %)	10.811	0.289
African American	15 (6.41%)	15 (3.84 %)	30 (4.8 %)	
Asian	25 (10.68%)	25 (6.39 %)	50 (8.0 %)	
Hispanic	24 (10.26%)	33 (8.44 %)	57 (9.1 %)	
Other	13 (5.56%)	18 (4.6 %)	31 (5.0 %)	
**Has health insurance**					
No	22 (9.4%)	32 (8.2 %)	54 (8.64 %)	2.513	0.642
Yes	207 (88.5%)	355 (90.8 %)	562 (89.92 %)	
No response	5 (2.1%)	4 (1.0%)	9 (1.44%)	
**Have regular physician**					
No	77 (32.9%)	88 (22.5%)	165 (26.4%)	8.808	0.003
Yes	141 (60.3%)	280 (71.6%)	421 (67.4%)	
No response	16 (6.8%)	23 (5.9%)	39 (6.2%)	
**Asked by physician to update family health history**					
Once	88 (37.6%)	232 (59.4%)	320 (51.2%)	28.452	<0.001
Twice	95 (40.6%)	101 (25.8%)	196 (31.4%)	
Three times	35 (15.0%)	35 (8.9%)	70 (11.2%)	
No response	16 (6.8%)	23 (5.9%)	39 (6.2%)	
**Responsibility to initiate family health history discussions**					
Mine	106 (45.3%)	155 (39.6%)	261 (41.8%)	13.948	0.175
Equal	66 (28.2%)	125 (32.0%)	191 (30.6%)	
Physician	46 (19.7%)	88 (22.5%)	134 (21.4%)	
No response	16 (6.8%)	23 (5.9%)	39 (6.2%)	
**Number of family members diagnosed^a^**					
Heart disease	0.530 (±0.787)	0.737 (±0.944)	0.659 (±0.894)	−2.813	0.005
Cancer	0.761 (±0.841)	1.001 (±0.966)	0.914 (±0.928)	−3.211	0.001
Diabetes	0.637 (±0.917)	0.698 (±0.839)	0.675 (±0.869)	−0.856	0.393
Obesity/overweight	0.523 (±0.950)	0.737 (±1.154)	0.658 (±1.087)	−2.357	0.019
**Number of other people diagnosed^a^**					
Heart disease	0.350 (±0.790)	0.310 (±0.726)	0.325 (±0.750)	0.660	0.509
Cancer	0.692 (±1.010)	0.865 (±1.083)	0.800 (±1.059)	−1.971	0.049
Diabetes	0.534 (±0.922)	0.637 (±0.993)	0.598 (±0.968)	−1.284	0.200
Obesity/overweight	0.513 (±0.955)	0.532 (±1.097)	0.525 (±1.045)	−0.221	0.825

*^a^t-tests and p-values reported for continuous variables*.

When comparing these variables by sex, as seen in the bivariate relationships provided in Table 1, a significantly larger proportion of female participants reported having a primary physician and being asked once by their physician’s office to update their family health history. On average, female participants reported having significantly more family members diagnosed with heart disease (*t* = −2.81, *p* = 0.005), cancer (*t* = −3.21, *p* = 0.001), and obesity/overweight (*t* = −2.36, *p* < 0.05) when compared to their male counterparts.

Individuals with whom participants have discussed their family health history, with whom they would discuss their family health history, and with whom they prefer to discuss their family health history are presented in Table 2. When asked with whom participants have discussed their family health history, 85.6% reported their parents, 50.1% their siblings, 43.7% their extended family, 39.4% their friends/peers, and 56.2% their physician/healthcare provider. Comparing these variables by sex, a significantly larger proportion of female participants reported having discussed their family health history with their parents, siblings, extended family, friends/peers, and physician/healthcare provider. Conversely, a significantly larger proportion of male participants reported having family health history discussions with no one. On average, female participants reported having discussed their family health history with more types of individuals (*t* = −5.12, *p* < 0.001).

**Table 2:** **Who college students have had, would have, and prefer to have family health history discussions with by sex (*n* = 625)**.

	Have discussed family health history				Would discuss family health history				Prefer to discuss family health history			
	Total Number of People: *t* = –5.118, *p* < 0.001				Total Number of People: *t* = –3.533, *p* < 0.001				Total Number of People: *t* = –2.796, *p* = 0.005			
	Male (*n* = 234)	Female (*n* = 391)	Total (*n* = 625)	*p*	Male (*n* = 234)	Female (*n* = 391)	Total (*n* = 625)	*p*	Male (*n* = 234)	Female (*n* = 391)	Total (*n* = 625)	*p*
Parents	186 (79.5%)	349 (89.3%)	535 (85.6%)	**	191 (81.6%)	354 (90.5%)	545 (87.2%)	**	191 (81.6%)	349 (89.3%)	540 (86.4%)	**
Siblings	98 (41.9%)	215 (55.0%)	313 (50.1%)	**	169 (72.2%)	309 (79.0%)	478 (76.5%)		139 (59.4%)	251 (64.2%)	390 (62.4%)	
Extended family	82 (35.0%)	191 (48.8%)	273 (43.7%)	**	137 (58.5%)	299 (76.5%)	436 (69.8%)	**	87 (37.2%)	180 (46.0%)	267 (42.7%)	*
Friends/peers	69 (29.5%)	177 (45.3%)	246 (39.4%)	**	130 (55.6%)	275 (70.3%)	405 (64.8%)	**	61 (26.1%)	156 (39.9%)	217 (34.7%)	**
Physician/healthcare provider	96 (41.0%)	255 (65.2%)	351 (56.2%)	**	170 (27.2%)	336 (53.8%)	506 (81.0%)	**	138 (59.0%)	272 (69.6%)	410 (65.6%)	**
Teachers/educators	18 (7.7%)	45 (11.5%)	63 (10.1%)		75 (32.1%)	160 (40.9%)	235 (37.6%)	*	18 (7.7%)	36 (9.2%)	54 (8.6%)	
Religious figure	8 (3.4%)	19 (4.9%)	27 (4.3%)		68 (29.1%)	133 (34%)	201 (32.2%)		23 (9.8%)	27 (6.9%)	50 (8.0%)	
Strangers	5 (2.1%)	8 (2.0%)	13 (2.1%)		29 (12.4%)	35 (9%)	64 (10.2%)		4 (1.7%)	5 (1.3%)	9 (1.4%)	
Other	9 (3.8%)	11 (2.8%)	20 (3.2%)		18 (7.7%)	26 (6.6%)	44 (7.0%)		10 (4.3%)	7 (1.8%)	17 (2.7%)	
No one	22 (9.4%)	10 (2.6%)	32 (5.1%)	**	11 (4.7%)	6 (1.5%)	17 (2.7%)	*	5 (2.1%)	4 (1.0%)	9 (1.4%)	

*p < 0.05; **p < 0.01.

*Values reported in this table represent the proportion of participants who “endorsed” each variable*.

When asked with whom participants would discuss their family health history, 87.2% reported their parents, 76.5% their siblings, 69.8% their extended family, 64.8% their friends/peers, 81.0% their physician/healthcare provider, and 37.6% teachers/educators. Comparing these variables by sex, a significantly larger proportion of female participants reported that they would discuss their family health history with their parents, extended family, friends/peers, physician/healthcare provider, and teachers/educators. Conversely, a significantly larger proportion of male participants reported that they would discuss their family health history with no one. On average, female participants reported that they would discuss their family health history with more types of individuals (*t* = −3.53, *p* < 0.001).

When asked with whom participants would prefer to discuss their family health history, 86.4% reported their parents, 65.6% their physician/healthcare provider, 62.4% their siblings, and 42.7% their extended family. Comparing these variables by sex, a significantly larger proportion of female participants reported that they would prefer to discuss their family health history with their parents, extended family, friends/peers, and physician/healthcare provider. On average, female participants reported that they would prefer to discuss their family health history with more types of individuals (*t* = −2.80, *p* = 0.005).

Factors that would influence participants to discuss their family health history, their perceived benefits of obtaining their family health history, and their perceived barriers to obtaining their family health history are presented in Table 3. When asked to select why they have discussed their family health history with others from a list of seven choices, participants’ top three reasons endorsed included: “a family member was diagnosed with a disease/health condition” (64.5%), “a friend/peer was diagnosed with a disease/health condition” (41.8%), and “an outside source made me aware of the disease/health condition” (38.7%). Comparing these responses by sex, a significantly larger proportion of female participants reported that they have discussed their family health history with others because: “I had symptoms of a disease/health condition,” “I was diagnosed with a disease/health condition,” “a family member was diagnosed with a disease/health condition,” and “a friend/peer was diagnosed with a disease/health condition.” Conversely, a significantly larger proportion of males reported “I do not discuss health issues with others,” when compared to their female counterparts. On average, females reported that they have discussed their family health history with others for significantly more reasons than their male counterparts (*t* = 25.12, *p* < 0.001).

**Table 3:** **Factors influencing family health history discussions by sex (*n* = 625)**.

	Male (*n* = 234)	Female (*n* = 391)	Total (*n* = 625)	X^2^/t	*p*
**Have discussed family health history because**				**25.119**	**<0.001**
I had symptoms of a disease/health condition	51 (21.8%)	163 (41.7%)	214 (34.2%)	25.729	<0.001
I was diagnosed with a disease/health condition	30 (12.8%)	96 (24.6%)	126 (20.2%)	12.518	<0.001
A family member was diagnosed with a disease/health condition	126 (53.8%)	277 (70.8%)	403 (64.5%)	18.467	<0.001
A friend/peer was diagnosed with a disease/health condition	85 (36.3%)	176 (45.0%)	261 (41.8%)	4.543	0.033
An outside source made me aware of the disease/health condition	80 (34.2%)	162 (41.4%)	242 (38.7%)	3.238	0.072
I do not discuss health issues with others	33 (14.1%)	28 (7.2%)	61 (9.8%)	8.009	0.005
Other	29 (12.4%)	32 (8.2%)	61 (9.8%)	2.945	0.086
**Would discuss family health history because**				**50.095**	**<0.001**
I had symptoms of a disease/health condition	164 (70.1%)	326 (83.4%)	490 (78.4%)	15.269	<0.001
I was diagnosed with a disease/health condition	159 (67.9%)	333 (85.2%)	492 (78.7%)	25.906	<0.001
A family member was diagnosed with a disease/health condition	157 (67.1%)	334 (85.4%)	491 (78.6%)	29.195	<0.001
A friend/peer was diagnosed with a disease/health condition	132 (56.4%)	303 (77.5%)	435 (69.6%)	30.755	<0.001
An outside source made me aware of the disease/health condition	95 (40.6%)	215 (55.0%)	310 (49.6%)	12.124	<0.001
I would not discuss health issues with others	17 (7.3%)	11 (2.8%)	28 (4.5%)	6.779	0.009
Other	30 (12.8%)	96 (24.6%)	126 (20.2%)	12.518	<0.001
**Would be motivated to obtain family health history because**				**59.752**	**<0.001**
I experience symptoms of a disease/health condition	183 (78.2%)	336 (85.9%)	519 (83.0%)	6.208	0.013
I am diagnosed with a disease/health condition	165 (70.5%)	330 (84.4%)	495 (79.2%)	17.135	<0.001
A friend is diagnosed with a disease/health condition	97 (41.5%)	216 (55.2%)	313 (50.1%)	11.135	0.001
A friend’s family member is diagnosed with a disease/health condition	78 (33.3%)	172 (44.0%)	250 (40.0%)	6.927	0.008
One of my biological parents are diagnosed with a disease/health condition	166 (70.9%)	332 (84.9%)	498 (79.7%)	17.646	<0.001
A sibling is diagnosed with a disease/health condition	160 (68.4%)	307 (78.5%)	467 (74.7%)	7.969	0.005
I reach an age that puts me at a higher risk of getting a disease/health condition	124 (53.0%)	277 (70.8%)	401 (64.2%)	20.290	<0.001
I learn about a disease/health condition in an educational setting	94 (40.2%)	225 (57.5%)	319 (51.0%)	17.683	<0.001
I learn that genetics are a risk factor in an educational setting	108 (46.2%)	252 (64.5%)	360(57.6%)	20.065	<0.001
I learn about a disease/health condition from a non-educational source	62 (26.5%)	153 (39.1%)	215 (34.4%)	10.356	0.001
I learn that genetics are a risk factor from a non-educational source	75 (32.1%)	186 (47.6%)	261 (41.8%)	14.496	<0.001
A friend tells me that knowing my family health history is important	66 (28.2%)	142 (36.3%)	208 (33.3%)	4.338	0.037
A family member tells me that knowing my history is important	101 (43.2%)	210 (53.7%)	311 (49.8%)	6.513	0.011
A religious figure tells me that knowing my history is important	49 (20.9%)	130 (33.2%)	179 (28.6%)	10.851	0.001
A teacher/professor/instructor tells me that knowing my history is important	70 (29.9%)	156 (39.9%)	226 (36.2%)	6.320	0.012
**Perceived benefits of obtaining family health history**				**21.285**	**0.006**
My physician can use my history to help with my disease/health condition diagnosis	207 (88.5%)	357 (91.3%)	564 (90.2%)	13.073	<0.001
My physician will use my history to give me personalized treatment recommendations	204 (87.2%)	358 (91.6%)	562 (89.2%)	6.893	0.075
Knowing my history will help me prevent diseases/health conditions that run in my family	203 (86.8%)	353 (90.3%)	556 (89.0%)	4.983	0.173
Knowing my history will help me know my risk for diseases/health conditions that run in my family	205 (87.6%)	360 (92.1%)	565 (90.4%)	7.720	0.052
Knowing my history is important when deciding to have children	181 (77.4%)	322 (82.4%)	503 (80.5%)	11.616	0.009
Knowing my history is important if I become ill	205 (87.6%)	353 (90.3%)	558 (89.3%)	19.178	<0.001
Knowing my history is important regardless of my health status	203 (86.8%)	354 (90.5%)	557 (89.1%)	9.800	0.020
**Barriers to personally obtaining family health history in the next 3 days because**				**10.022**	**0.692**
Do not know how to obtain my history	69 (29.5%)	75 (19.2%)	144 (23.0%)	8.768	0.003
Do not know who to ask to obtain my history	53 (22.6%)	61 (15.6%)	114 (18.2%)	4.877	0.027
Do not know what questions to ask to obtain my history	75 (32.1%)	109 (27.9%)	184 (29.4%)	1.228	0.268
Do not think anyone knows my history	19 (8.1%)	31 (7.9%)	50 (8.0%)	0.007	0.932
Adopted and do not know my biological parents	1 (0.4%)	9 (2.3%)	10 (1.6%)	3.267	0.071
Do not have contact with one or both of my biological parents	10 (4.3%)	31 (7.9%)	41 (6.6%)	3.190	0.074
Do not live close to the people I need to discuss history with	40 (17.1%)	61 (15.6%)	101 (16.2%)	0.241	0.624
Those with information about my history are deceased	10 (4.3%)	26 (6.6%)	36 (5.8%)	1.523	0.217
The exact cause of death for one or more of my relatives is unknown	12 (5.1%)	44 (11.3%)	56 (9.0%)	6.733	0.009
My biological father would not be able to tell me about my history	12 (5.1%)	27 (6.9%)	39 (6.2%)	0.790	0.374
My biological mother would not be able to tell me about my history	11 (4.7%)	7 (1.8%)	18 (2.9%)	4.434	0.035
Feel uncomfortable asking about my history	15 (6.4%)	25 (6.4%)	40 (6.4%)	0.000	0.994
Worry about confidentiality when asking about my history	8 (3.4%)	9 (2.3%)	17 (2.7%)	0.690	0.406
Do not feel close to anyone enough to ask about my history	6 (2.6%)	7 (1.8%)	13 (2.1%)	0.430	0.512
Obtaining my history may make me depressed	21 (9.0%)	39 (10.0%)	60 (9.6%)	0.169	0.681
Obtaining my history may keep me from getting insurance coverage	8 (3.4%)	6 (1.5%)	14 (2.2%)	2.374	0.123
Obtaining my history is very time consuming	59 (25.2%)	108 (27.6%)	167 (26.7%)	0.433	0.510
Obtaining my history will make me feel destined to get diseases that run in my family	34 (14.5%)	67 (17.1%)	101 (16.2%)	0.734	0.392
Obtaining my history will make me feel helpless	9 (3.8%)	25 (6.4%)	34 (5.4%)	1.847	0.174

*t-tests and p-values reported for continuous variables (i.e., total number endorsed) in bold*.

*Values reported in this table represent the proportion of participants who “endorsed” each variable*.

When asked to select why they would discuss their family health history with others from a list of seven choices, participants’ top three reasons endorsed included: “I had symptoms of a disease/health condition” (78.4%), “I was diagnosed with a disease/health condition” (78.7%), and “a family member was diagnosed with a disease/health condition” (78.6%). Comparing these responses by sex, females reported that they would discuss their family history with others for significantly more reasons than their male counterparts (*t* = 50.10, *p* < 0.001). With the exception of the response choice “I do not discuss health issues with others,” a significantly larger proportion of female participants reported that they would discuss their family health history with others for the other six response options.

When asked to select what would motivate participants to obtain their family health history from a list of 15 choices, participants’ top three reasons endorsed included: “I experience symptoms of a disease/health condition” (83.0%), “I am diagnosed with a disease/health condition” (79.2%), and “one of my biological parents was diagnosed with a disease/health condition” (79.7%). Additionally, 64.2 and 57.6% reported that they would be motivated to obtain their family health history because “I reach an age that puts me at higher risk of getting a disease/health condition” and “I learn that genetics are a risk factor in an educational setting,” respectively. Comparing these responses by sex, females reported that they would be motivated to obtain their family health history for significantly more reasons than their male counterparts (*t* = 59.75, *p* < 0.001). A significantly larger proportion of female participants reported that they would be motivated to obtain their family health history for all 15 response options when compared to their male counterparts.

When asked to select what they perceive to be benefits of obtaining their family health history from a list of seven response choices, participants’ top two responses included: “My physician can use my family health history to help with my disease/health condition diagnosis” (90.2%), and “Knowing my family history will help me know my risk for disease/health conditions that run in my family” (90.4%). Comparing these responses by sex, a significantly larger proportion of female participants reported benefits of obtaining their family health history to be: “My physician can use my family health history to help with my disease/health condition diagnosis,” “Knowing my history is important when deciding to have children,” “Knowing my history is important if I become ill,” and “Knowing my history is important regardless of my health status.” On average, females reported more benefits to obtaining their family health history than their male counterparts (*t* = 21.29, *p* = 0.006).

When asked to select what they perceive to be barriers to obtaining their family health history within the next 3 days from a list of 19 response choices, participants’ top five responses included: “Don’t know what questions to ask to obtain my history” (29.4%), “Obtaining my history is very time consuming” (26.7%), “Don’t know how to obtain my history” (23.0%), “Don’t live close to the people I need to discuss history with” (16.2%), and “Obtaining my history will make me feel destined to get diseases that run in my family” (16.2%). Comparing these responses by sex, a significantly larger proportion of male participants reported the following barriers to obtaining their family health history within the next 3 days: “Don’t know how to obtain my history,” “Don’t know who to ask to obtain my history,” and “My biological mother would not be able to tell me about my history.” Conversely, a significantly larger proportion of female participants reported “The exact cause of death for one or more of my relatives is unknown.”

## Discussion

### Females Willingness to Discuss Family Health History

Our findings revealed that females reported a higher likelihood of discussing family health history compared to their male counterparts. As demonstrated in Table 2, females were significantly more likely to have discussed family health history with a wide range of people relative to their male counterparts. These same relationships persisted in terms of with whom females would discuss family health history. Females exhibited higher comfort levels for discussing their family health history with friends and peers relative to their male counterparts. Further, a larger proportion of females reported that they would rather discuss family health history with their friends and peers than with their physicians. Previous studies have confirmed the importance of these friend and peer relationships in developing risk perceptions of disease (22), and our results add to these findings by extending the importance of friends and peers to the role of confidant for family health history discussions, especially among females.

Identifying why females are more likely to discuss family health history may be partly answered in the results presented within Table 3. Females may have been more likely than their male counterparts to have discussions about their family health history because they had symptoms of disease or had a relative who was diagnosed with a disease. These findings were similar when females were asked with whom they would share their family health history. One possible interpretation is that females are more likely than their male counterparts to recognize disease-related symptoms and visit their physicians when they experience symptoms that concern them or when they witness a diagnosis of a family member. However, as demonstrated in Table 1, when females visited their physicians, they were asked by their physicians to update their family health history less frequently than their male counterparts. This finding appears to reflect either biases of physicians to request family health history information for certain patients, an issue of motivation, or differences in perceived importance for family health history between males and females in this study. Or, because a significantly larger proportion of males reported discussing their family health history with “no one” and perceived more barriers to obtaining their family health history, males may be asked by their physicians to update their family health history more often because they visit the physician less frequently (see Table 1) or have less complete/detailed health records.

The findings about sex differences in interpersonal discussion on family health history also have implications for communication research and theory. That females reported a higher likelihood of discussing family health history compared to their male counterparts is generally supportive of research contending that health communication and health information processes vary by sex (8), with women tending to have higher levels than men (9, 10, 23). These findings also support research reporting that women have higher levels of utilization of healthcare services (22). Not only do women tend to have higher levels of access to health services but also the principal responsibility for the healthcare and health services of their families (24, 25). However, sex-based differences observed in this study suggest the need to intentionally target men with regard to interventions (e.g., educational, awareness, clinical) intending to increase FHH attainment and related discussions. Further research may be required to adequately identify potential strategies that would be effective for men.

### Is Motivation to Act Representative of Optimistic Bias?

Table 3 illustrates the various motivations to obtain family health history among the college students in this study. Females were significantly more motivated than their male counterparts to obtain their family health history based on the situations posed to them in the questionnaire. However, two sex-based differences are particularly meaningful. First, females were significantly more likely than males to obtain their family health history if their parents were diagnosed with a health condition. This suggests that females were more likely to recognize the importance of family health history to their own health when a direct family member was diagnosed. And, this perception of the importance of family health history would also motivate them to act. Second, females were significantly more likely than their male counterparts to report being likely to obtain their family health history when they “reached an age that put them at a higher risk of developing a health condition.” Recognizing the statistical likelihood of certain diseases at certain points in the life course provides suggestive evidence that females have a lower optimistic bias than their male counterparts.

These results, again, underscore the predominant role of women in the context of health and healthcare. Women are more motivated than men to communicate about family health history, which is in-line with their having higher levels of utilization of healthcare services (26) and their having the principal responsibility for the healthcare and health services of their families (24, 25). The current study, thus, has determined that women have higher levels of interpersonal communication about family health history and higher levels of motivation for such interpersonal communication. It is interesting that such sex-based roles appear to be engrained as early as the college years. Moreover, these two patterns in interpersonal communication and motivation for such interpersonal communication, when considered in tandem, are generally with research on uses and gratifications, which has found that people with higher levels of motivation for media use tend to have higher levels of actual media use (18, 27). In this light, interpersonal communication about family health history can be conceived to be an active and goal-oriented process.

### Barriers Affect both Sexes

Table 3 reports the perceived barriers to obtaining family health history among college students. These findings highlight that although college students can be informed how important it is to obtain their family health history, a multitude of factors exist to hinder their ability to successfully acquire such information and use it appropriately during life transitions. Therefore, as discussed briefly below, a variety of communication-driven interventions can be used to increase knowledge and skills associated with obtaining family health information in a timely manner, so it can be used properly. These are the only set of analyses in this study that do not represent easily identifiable differences between males and females. Two of the noteworthy non-statistically significant observations between sexes included the barriers of finding time to obtain their family health history and knowing which questions to ask to document their family health history.

Although a high percentage of participants reported discussing their family health history with their parent (85.6%), substantially fewer participants reported discussing this information with physicians (56.2%), which may represent the lack of priority college students place on sharing their family health history. While only 56.2% of participants have discussed FHH with their physician, substantially more stated they would discuss FHH with their physician (81.0%). This finding suggests that physicians are respected and well placed to address FHH within consultations in clinical settings. A possible solution to increase FHH discussions with physicians could be to require students to have this information on file with their university’s student health service to make them eligible for class registration. Similar to required immunization programs, a required family health history that is certified by students and school health officials could build a foundation enabling college students to value obtaining their family health history. Alongside these potential university requirements, educational interventions could be offered as high school students’ transition to college through incoming freshman seminars or other university-sanctioned activities/events. These efforts might highlight the importance of obtaining family health history, increase documenting family health history compliance, and also raise awareness about protective lifestyle behaviors.

Knowing which questions should be asked to obtain family health history is a barrier that can be overcome with educational interventions. Both student health services and primary care physicians could benefit from the development of tools to guide college students through the process of obtaining family health history (e.g., see http://www.hhs.gov/familyhistory/portrait/index.html). Such tools could also be utilized by parents when preparing their children for college. Thus, a mentality could be developed reinforcing that leaving home with their family health history is just as vital as leaving with their immunization records, housing assignments, or first credit card.

The general constant levels of perceived barriers among male and female students relates to HBM. HBM posits that a person’s likelihood of behavioral change results from his/her perceptions of benefits versus perceptions of barriers (17). While the current study documents sex differences in motivation (e.g., utility) and interpersonal communication (which is an information-seeking action), there is no evidence of sex differences in beliefs, such as those pertinent to barriers. Thus, it may be that, in the context of family health history, beliefs are not an underlying mechanism that can help explain sex differences in motivation and interpersonal discussion.

### Limitations

Our study is primarily descriptive and utilized self-reported data. Second, our study utilized a cross-sectional design from a single college campus. As such, results should not be generalized beyond this sample. Third, future studies should be conducted that have a longitudinal focus. Studying college students over time may be valuable to examine how their perceptions change during their college experience, and what motivates them to overcome barriers to obtaining a family health history. Fourth, the actual existence and quality of family relationships were not measured, thus it was difficult to ascertain the context of motivations and barriers to obtaining (and discussing) family health history.

## Conclusion

Unlike previous studies, we did not seek to emphasize the optimistic bias or discuss intentions to obtain family health history based solely on a single disease. This study suggests that there are differences in the ways males and females discuss and share their family health history. We revealed that females have discussed and would discuss their family health history with a wider range of people than their male counterparts. This study identified that when optimistic bias is a concern, it likely relates to the motivational aspect of obtaining family health history, and there is a significant difference in such motivation based on sex. Finally, this study identified possible strategies in which common barriers to obtaining family health history can be overcome. These findings have implications to inform public health officials and health promotion researchers during the formulation of campus and community policies. Although it is critical to tell college students that family health history is important, they must be enabled to successfully obtain this information and use it at appropriate life transitions. Furthermore, these findings have implications for research on the predictive processes of health information-seeking and -related communication actions. This study provides general evidence of related communication patterns and how they may vary according to one demographic, sex. Future research is encouraged to continue to explore the role that this demographic variable may have in different communication models and different health contexts.

## Conflict of Interest Statement

The authors declare that the research was conducted in the absence of any commercial or financial relationships that could be construed as a potential conflict of interest.
